# The compound losartan cream inhibits scar formation via TGF-β/Smad pathway

**DOI:** 10.1038/s41598-022-17686-y

**Published:** 2022-08-22

**Authors:** Wan-Yi Zhao, Li-Yun Zhang, Zheng-Cai Wang, Qing-Qing Fang, Xiao-Feng Wang, Yong-Zhong Du, Bang-Hui Shi, Dong Lou, Gui-Da Xuan, Wei-Qiang Tan

**Affiliations:** 1 Department of Plastic Surgery, Sir Run Run Shaw Hospital, Zhejiang University School of Medicine, 3 East Qingchun Road, Hangzhou, 310016 Zhejiang Province People’s Republic of China; 2 School of Medicine, Zhejiang University City College, 51 Huzhou Street, Hangzhou, 310015 Zhejiang Province People’s Republic of China; 3 Institute of Pharmaceutics, College of Pharmaceutical Sciences, Zhejiang University, Hangzhou, Zhejiang Province People’s Republic of China; 4 Department of Plastic Surgery, The Fourth Affiliated Hospital, Zhejiang University School of Medicine, Yiwu, Zhejiang Province People’s Republic of China

**Keywords:** Trauma, Drug development, Preclinical research

## Abstract

The role of angiotensin receptor blocker in wound healing and cutaneous fibrosis has become a hotspot in recent years. We have developed a losartan cream that is comparable to triamcinolone ointment in inhibiting scarring. Considering the effects of chitosan and asiaticoside on wound healing and scarring, we added them to the losartan cream this time and improved the formula, expecting to get a better anti-scarring effect. The effects of creams were investigated on mouse scar model with triamcinolone ointment, onion extract gel, and commercial asiaticoside cream set as positive controls. A preliminary exploration of the mechanism involved in TGF-β/Smad pathway was performed in vivo and in vitro. With all results of anti-scarring, the compound losartan cream (containing chitosan, asiaticoside, and losartan) shows the best effect, followed by the chitosan asiaticoside cream. The treatment of the compound losartan cream inhibited expression of TGF-β1, collagen, and Smads, and decreased phosphorylation of Smad in vivo. These inhibitory effects were also confirmed in vitro. Our findings indicated that the compound losartan cream could inhibit scarring via TGF-β/Smad pathway. This cream might be an effective option for scar treatment.

## Introduction

Scarring of the skin, normally caused by surgery and burns, is a serious global medical problem that cannot be ignored. In the developed world, an estimated 100 million patients per year have scars as a result of operations^1,2^. Patients with scars, especially children, may suffer from physical dysfunction and even psychological diseases^3,4^. Thus, it is important to inhibit scar formation in its early stage.

The treatment of scars is normally divided into surgical and non-surgical methods. Surgical revision is performed to correct the anatomic structures, reduce the tension, and hide these mature scars^5–7^. Non-surgical treatment includes topical treatments, laser, injection, pressure therapy, etc.^7^ These therapies all have limited efficacy and cannot completely eliminate scarring^7,8^.

TGF-β/Smad pathway is the most classic signaling pathway that contributes to scarring. Renin-Angiotensin System (RAS), which is closely related to TGF-β/Smad pathway, is also shown to be involved in fibrosis in various organs^9–11^. A complete RAS also exists in the skin tissue^12^, and participates in scar formation^13^. Angiotensin II can upregulate TGF-β expression by activating RAS^14^. It has been proved that angiotensin converting enzyme inhibitor (ACEI) and angiotensin receptor blocker (ARB) could inhibit scar formation through TGF-β/Smad pathway^15^. Based on this published data, we preliminarily developed ARB cream containing losartan to avoid the side effects, such as hypotension, in our previous research, and verified the anti-scarring ability of them^16^.

Some natural drugs may also have the potential to inhibit scar formation. Chitosan and asiaticoside, as natural materials, have been proven to have positive effects of promoting wound healing, of which the mechanisms are related to the inhibition on TGF-β pathway^17–21^. Chitosan is a linear amino polysaccharide consisting of glucosamine and N-acetyl glucosamine units which are linked by β (1–4) glycosidic bonds^22^. Chitosan has been used for wound treatment because of its excellent biocompatibility, biodegradability, antibacterial properties, and other biological properties^23^. Numerous studies have shown that chitosan can promote wound healing and reduce scar formation^24,25^. Asiaticoside is a kind of saponin component extracted from *Centella asiatica*. It has been verified that asiaticoside has a variety of biological effects, including anti-inflammatory^26^ and antioxidant^27^. Asiaticoside was proven to reduce fibroblast proliferation and inhibit the expression of type I as well as type III collagen in a time- and dose-dependent manner^28^, and it has been used for scar treatment for many years.

Since all of chitosan, asiaticoside, and ARB have direct or indirect moderating effect on TGF-β signaling pathway, the combination of them may maximize effect. We developed a kind of ARB cream (containing losartan) with anti-scarring effect in our previous study, however, the effect of the ARB cream had no significant difference compared with triamcinolone ointment, the positive control^16^. Therefore, we combined chitosan, asiaticoside, and losartan in this study to develop a more efficient compound cream with better inhibitory effect on scarring and explored the mechanism preliminarily.

## Materials and methods

### Creams preparation and permeation assay

#### Materials resources

*Centella asiatica* extract (containing approximately 80% asiaticoside) was obtained from Guilin Chuang Ying Biotechnology Co., Ltd, (Guilin, Guangxi, China). Losartan was obtained from Zhejiang Tianyu Pharmaceutical Co., Ltd, (Taizhou, Zhejiang, China). High (MW ~ 800,000) and low (MW ~ 3000) molecular weight chitosan (deacetylation degree of 95%) were obtained from Zhejiang Golden-Shell Pharmaceutical Co., Ltd, (Yuhuan, Zhejiang, China). All other materials used to produce the cream base, the chitosan asiaticoside cream (the CA cream, containing chitosan and asiaticoside), the compound losartan cream (the CAL cream, containing chitosan, asiaticoside, and losartan), and the losartan cream were obtained from Hangzhou Meikezi biotechnology Co., Ltd (Hangzhou, Zhejiang, China).

#### Creams preparation

The formula of the creams and the materials resources are listed in Table 1. To make the cream base, the CA cream, the CAL cream, and the losartan cream, materials (except urea and PEHG) were added into water phase and oil phase respectively. The mixture ratio of the two kinds of chitosan mentioned in this study was 1:1, which was determined by our previous study and patent^29,30^. Two phases were stirred and heated to 80 °C to dissolve completely. Urea was added into the water phase with 1% concentration (w/w) after cooling two phases to 40 °C. Then two phases were mixed, and PEHG was added to the mixture with 0.4% concentration (w/w). Finally, after cooling and emulsifying, the creams were prepared.

**Table 1:** Cream formula table.

	Materials	Weight percentage (%)	Content in 200 g cream (g)
Water phase	ddH_2_O	61.45	122.9
	Glycerin	5	10
	Trehalose	2	4
	EDTA-2Na	0.05	0.1
	Methylparaben	0.2	0.4
	Urea	1	2
	***Centella asiatica* extract**	**2.5**	**5**
	**Losartan**	**5**	**10**
	**Chitosan (MW ~ 800,000)**	**2**	**4**
	**Chitosan (MW ~ 3000)**	**2**	**4**
Oil phase	A25 (ceteareth-25)	2	4
	A170 (stearin)	1	2
	GMS (glyceryl monostearate)	1	2
	Aliphatic alcohol	5	10
	2-EHP (Isooctyl palmitate)	7	14
	GTCC (caprylic/capric triglyceride)	3	6
	DC200 (simethicone)	1.5	3
	D5 (decamethylcyclopentasiloxane)	5	10
	Artificial squalane	3	6
	Shea butter	1	2
	DL-alpha-tocopherol acetate	0.2	0.4
	Propylparaben	0.1	0.2
Preservative	PEHG	0.4	0.8

Urea was added into water phase after cooling to 40 °C, and PEHG was added after two phases were mixed. The cream base contained all materials above except *Centella asiatica* extract, losartan and two kinds of chitosan; the chitosan asiaticoside cream (the CA cream) contained all materials above except losartan; the compound losartan cream (the CAL cream) contained all materials above; the losartan cream contained all materials above except *Centella asiatica* extract and two kinds of chitosan. The 4 drugs used in this study are in bold.

#### Transdermal permeation assay

The investigation of drug transdermal absorption was carried out by transdermal permeation experiment, high performance liquid chromatography (HPLC), and fluorescence labeling. The cream base was used for control.

Suckling pigskin (Meisheng Trading, Chongqing, China) was used to simulate the human skin in this experiment. The pigskin was stored at the -80 °C refrigerator to maintain biological activity. Before the experiment, pigskin was cut into square pieces (side length = 3.0 cm) at room temperature. The pigskin pieces were fixed with pins, 0.48 ml cream was applied to each piece with a syringe. After being gently massaged for 5 min, the pigskin pieces were wiped with gauze soaked in normal saline and dried with absorbent paper.

The residual cream in the pigskin was separated by ultrasonic cleaner and extracted with special mobile phases and methyl alcohol. The mobile phase of losartan was acetonitrile–phosphate buffer (40:60, v/v)^31^, and the mobile phase of asiaticoside was acetonitrile-2 mmol/L β-cyclodextrin (24:76, v/v)^32^. The wiped pigskin pieces were cut into small square pieces (side length = 1 mm) and extracted in the ultrasonic cleaner using 10 ml methyl alcohol at 35 °C for 2 h. After that, a 10 ml mobile phase was added for another 2 h extraction. The supernatant was collected for HPLC assay after centrifugation (20 min, 300 rpm) and filtration (used 0.25 μm filter membrane). The drug extractions of creams were obtained through the same procedure with 0.48 ml creams respectively.

#### HPLC

HPLC was performed in a self-filled C_18_ column (150 mm × 4.5 mm, 5 μm) at room temperature with mobile phases mentioned above at a flow rate of 1 ml min^−1^. Losartan or asiaticoside was diluted with different concentrations using the mobile phase to prepare a standard solution. Drug transdermal absorption was evaluated from the peak area detected at 256 nm (losartan) and 205 nm (asiaticoside).

#### Fluorescence labeling and transdermal observation

Fluorescein isothiocyanate (FITC) was added into two kinds of chitosan (MW ~ 3000 and MW ~ 800,000) solution and were stirred overnight in a 1:2 mol ratio respectively. After overnight reaction in the dark at ambient temperature, the unreacted FITC was separated overnight by dialysis. Finally, creams were made from these two kinds of FITC-labeled chitosan.

Transdermal permeation was performed as above. OCT-embedded pigskin pieces were sectioned into 5.0 μm-thick histological slides and photographed using ZEISS AXIO Observer A1.

### Effects and mechanism investigation of creams in vivo

#### Mouse scar model

Animal welfare and experimental procedures were carried out in accordance with the ARRIVE guidelines and were approved by the Ethics Committee of Sir Run Run Shaw Hospital, School of Medicine, Zhejiang University. The mouse scar model was described in our previous research^15,16,33^. A total of 80 male C57BL/6 mice (average weight 25 g) were used in this study.

The C57BL/6 mice were anaesthetized with inhalational isoflurane (3% in oxygen for induction, 1.5% in oxygen for maintenance) using a rodent anesthesia machine (RWD Life Science, Shenzhen, Guangdong, China). The hair on the backs of the mice was removed. After asepsis with 70% ethanol, two rectangular skin pieces (1.5 cm × 0.2 cm) parallel to but 0.4 cm away from midline with thickness comprising the entire skin layers (panniculus carnosus was included) were excised on both sides of the back. After the scab fell off (about 12–14 days after surgery), the pictures of scars were taken every 2 days, and the scar widths were measured by Image J. Considering the differences in width within one scar, the measurements were taken at three locations on each side, and the average of scar widths on both sides was recorded as a data sample. Finally, the post-treatment scar ratio was calculated and recorded.

#### Grouping design and treatment in vivo

The mice were randomly divided into 8 groups (n = 10 in each group) and treated with corresponding creams. Three classic commercially available creams, which could inhibit scar formation through pharmacological action, were chosen as positive controls. They were Compound Heparin Sodium and Allantoin Gel (which contained onion extract) (Contractubex®, Merz Pharma GmbH&Co. KGaA, Frankfurt am Main, Germany), Triamcinolone Acetonide Acetate Urea Ointment (Fuyuan pharmaceutical Co. Ltd, Xuancheng, Anhui, China), and *Centella* Triterpenes Cream (Shanghai Shyndec Pharmaceutical Co. Ltd, Shanghai, China).

The mice had cream applied (0.04 ml of each side) on the scars every day after the scab falling off (14 days after surgery in this study). The remaining cream was wiped off with gauze soaked in saline after massaging for 5 min. 28 days after operation, the scars were harvested for histopathological assay, western blot, and RT-qPCR. Five mice in each group were randomly selected for histological analysis, and the other five were used for WB and RT-qPCR. After that, the mice were anaesthetized with inhalational isoflurane and sacrificed by cervical dislocation.

#### Histopathological analysis

Paraffin-embedded tissue samples were sectioned in 5.0 μm-thick histological slides. After that, hematoxylin–eosin (HE) staining, Masson's trichrome staining, and immunohistochemical staining were performed. The primary antibodies of TGF-β1, collagen I, and collagen III used in immunohistochemical analysis were purchased from Proteintech (Rosemont, USA). The dilution ratios of antibodies are shown in Table 2. The sections were scanned with Olympus VS120 Virtual Slide Microscope (Tokyo, Japan). The results of immunohistochemical staining were measured and recorded by Image J.

**Table 2:** Antibodies used in western blot and IHC.

Experiment	Antibody	Company	Address	RRID	Cat#	Dilution ratio
Western blot	Phospho-Smad2 (Ser465/467) antibody	Cell Signaling Technology	Danvers, MA, USA	AB_331673	3108	1:250
	Smad2 antibody	Cell Signaling Technology	Danvers, MA, USA	AB_10626777	5339	1:500
	Anti-Smad3 (phospho S423 + S425) antibody	Abcam	Cambridge, England	AB_882596	ab52903	1:2000
	Anti-Smad3 antibody	Abcam	Cambridge, England	AB_777979	ab40854	1:1000
	TGF-β1 antibody	Abcam	Cambridge, England	AB_10562492	ab92486	1:125
	Smad4 antibody	Santa Cruz Biotechnology	Dallas, Texas	AB_627905	sc-7966	1:400
	GAPDH Mouse Monoclonal antibody	Proteintech	Rosemont, CA, USA	AB_2107436	60004–1-Ig	1:5000
	HRP-conjugated Affinipure Goat Anti-Mouse IgG(H + L)	Proteintech	Rosemont, CA, USA	AB_2722565	SA00001-1	1:5000
	HRP-conjugated Affinipure Goat Anti-Rabbit IgG(H + L)	Proteintech	Rosemont, CA, USA	AB_2722564	SA00001-2	1:5000
IHC	TGF-β1 antibody	Proteintech	Rosemont, CA, USA	AB_2811115	21898–1-AP	1:400
	Collagen I antibody	Proteintech	Rosemont, CA, USA	AB_2082037	14695–1-AP	1:1000
	Collagen III antibody	Proteintech	Rosemont, CA, USA	AB_2879158	22734–1-AP	1:1000

#### Molecular biological analysis

Western blot was performed for protein detection. Samples were homogenized in RIPA buffer mixed with 2% protease inhibitor cocktail, 1% PMSF and 1% phosphorylase inhibitor. The proteins were separated by 10% sodium dodecyl sulphate–polyacrylamide gel electrophoresis (SDS-PAGE) and transferred to a polyvinylidene fluoride (PVDF) membrane. After that, the proteins were probed with appropriate primary antibodies at 4 °C overnight and then secondary antibodies at room temperature for 1 h. The Enhanced Chemiluminescence (ECL) and image acquisition were performed using a Fijifilm LAS-4000 Super CCD Remote Control Science Imaging System (GE Healthcare Bio-Sciences, Pittsburgh, PA, USA). Images were analysis by Image J. PageRuler™ Prestained Protein Ladder was obtained from Thermo Scientific, Scotts Valley, CA, USA. Other reagents used in western blot were provided by Proteintech, Rosemont, CA, USA. The details of primary and secondary antibodies are listed in Table 2. The western blot images were from different regions of different gels due to the similar molecular weight of the detected proteins, and all the western blot results were obtained under the same experimental conditions. The original images are presented in Supplementary Figures. Full-length images could not be shown because the PVDF membranes were cut to appropriate size before hybridization with different antibodies.

RT-qPCR was performed as follows for mRNA analysis. Total RNA was extracted using TaKaRa MiniBEST Universal RNA Extraction Kit (TaKaRa, Kusatsu, Shiga, Japan). The purified RNA (50–100 ng) was reverse transcribed using PrimeScript™ RT Master Mix (Perfect Real Time) (TaKaRa, Kusatsu, Shichi, Japan). The gene expression intensity was detected on LightCycler® 480 System (Roche Life Science, Indianapolis, IN, USA) using TB Green® Premix Ex Taq™ II (Tli RNaseH Plus) (TaKaRa, Kusatsu, Shichi, Japan). The relative expression levels of target genes were normalized by concurrently measured GAPDH mRNA levels. Fold-changes were calculated with 2^−ΔΔCt^ method. The primers were provided by TsingKe biotechnology Co., Ltd, Beijing, China. The primer sequences are listed in Table 3.

**Table 3:** The primer sequences used in quantitative RT-qPCR.

mRNA	The primer sequences	
TGF-β1	Sense	CTCCCGTGGCTTCTAGTGC
	Antisense	GCCTTAGTTTGGACAGGATCTG
Smad2	Sense	AAGCCATCACCACTCAGAATTG
	Antisense	CACTGATCTACCGTATTTGCTGT
Smad3	Sense	CACGCAGAACGTGAACACC
	Antisense	GGCAGTAGATAACGTGAGGGA
Smad4	Sense	ACACCAACAAGTAACGATGCC
	Antisense	GCAAAGGTTTCACTTTCCCCA
Collagen I	Sense	GCTCCTCTTAGGGGCCACT
	Antisense	CCACGTCTCACCATTGGGG
Collagen III	Sense	CTGTAACATGGAAACTGGGGAAA
	Antisense	CCATAGCTGAACTGAAAACCACC
GAPDH	Sense	AGGTCGGTGTGAACGGATTTG
	Antisense	TGTAGACCATGTAGTTGAGGTCA

### Effects and mechanism investigation of drugs in vitro

#### Cell culture

NIH3T3 fibroblasts (ATCC Cat# CRL-6442, RRID: CVCL_0594) were cultured in Dulbecco's Modified Eagle Medium (DMEM) (Gibco, MD, USA) containing 10% (v/v) fetal bovine serum (FBS), 1.5 g L^−1^ sodium bicarbonate, 4.5 g L^−1^ glucose, and 4 mM L-glutamine. Cytotoxicity of the drugs (mixture of chitosan, *Centella asiatica* extract and losartan) was detected by Cell Counting Kit-8 (CCK-8; Biosharp, Hefei, China).

#### Drug stimulation experiment in vitro

NIH3T3 cells at exponential growth period were seeded in 6-well plates with density of 1 × 10^5^ cells per well and cultured in DMEM containing 1% FBS for 24 h. Then the culture medium was replaced with DMEM containing 1% FBS and 1 ng ml^−1^ TGF-β1^15^. The CCK-8 assay was first carried out to detect the toxicity of different drugs to cells. Then the final concentration of the drugs used for stimulation was determined. Drug concentrations applied finally were as follows: *Centella asiatica* extract: 25 ng ml^−1^; losartan: 50 ng ml^−1^; chitosan: 40 ng ml^−1^. Corresponding drugs were added to different groups. TGF-β1 (Sigma, MO, USA) was used to simulate the traumatic stimulus^15^. Chitosan added into culture medium was the mixture of high-low molecular weight chitosan (1:1, w/w). NIH3T3 cells were cultured with drugs for another 24 h and then collected for western blot as well as RT-qPCR. The culture medium was collected for collagen detection.

#### Molecular biological analysis with cells

The culture medium of NIH3T3 cells was collected for collagen detection after drug stimulation. Sirius Red Total Collagen Detection Kit (Chondrex, WA, USA) was used for detection. Each sample had 2 replicate wells. The absorbance was detected at 520 nm, and the concentrations of samples were calculated according to the standard curve.

Western blot and RT-qPCR were performed as described in vivo.

### Statistical analysis

Data were analyzed with the SPSS (IBM-SPSS Inc., Chicago, IL, USA; version 22.0 for Windows) using one-way analysis of variance (ANOVA) method followed by post hoc Tukey. Values were expressed as mean ± SD. The value of *P* < 0.05 was considered significant. Graphs were drawn using GraphPad Prism (LaJolla, CA, USA; version 7.0 for Windows).

## Results

### Creams have efficient transdermal ability

**Figure 1:**
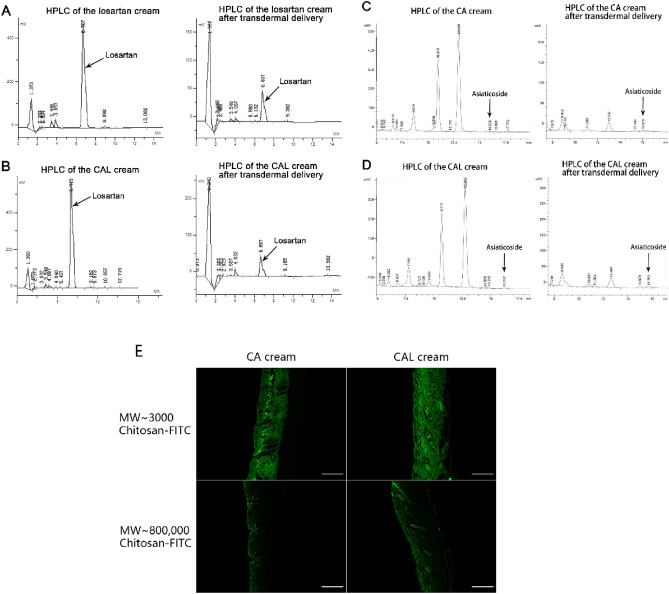
The transdermal penetration assays of creams. (**A**) The HPLC result of the losartan cream and the one after transdermal penetration (to detect losartan). (**B**) The HPLC result of the CAL cream and the one after transdermal penetration (to detect losartan). (**C**) The HPLC result of the CA cream and the one after transdermal penetration (to detect asiaticoside). (**D**) The HPLC result of the CAL cream and the one after transdermal penetration (to detect asiaticoside). (**E**) The transdermal penetration results of fluorescence labeled CA cream and CAL cream. The scale bar = 100 μm.

We developed the chitosan asiaticoside cream (the CA cream, containing chitosan and asiaticoside), the compound losartan cream (the CAL cream, containing chitosan, asiaticoside, and losartan), and the losartan cream according to Table 1, and these creams were test creams. The cream base was also made as negative control. HPLC was used to detect the transdermal permeation of losartan and asiaticoside, while fluorescence labeling was used in the transdermal permeation test of chitosan. The calculation results of HPLC are shown in Table 4, and the complete figures of HPLC and fluorescence labeling are shown in Fig. 1. According to the results, after 5 min of massage, losartan, asiaticoside, and chitosan were partially delivered into the skin. It was worth mentioning that the high molecular weight chitosan (MW ~ 800,000) was concentrated in the epidermis and the pores. This part of chitosan might play a moisturizing role^34^.

**Table 4:** HPLC results of creams and transdermal drugs.

Cream	The losartan cream		The CA cream		The CAL cream	
Drug	Losartan	Asiaticoside	Losartan	Asiaticoside	Losartan	Asiaticoside
Drug content	4.34 ± 0.04%	–	–	2.34 ± 0.03%	4.86 ± 0.03%	2.24 ± 0.03%
Transdermal permeation ratio	3.82 ± 0.03%	–	–	4.72 ± 0.03%	1.50 ± 0.03%	3.45 ± 0.04%

### Medicated creams reduce cutaneous scar formation in a mouse model

In order to investigate the effect of creams, we applied them to mouse scar models after scar formation. The treatment lasted 14 days.

**Figure 2:**
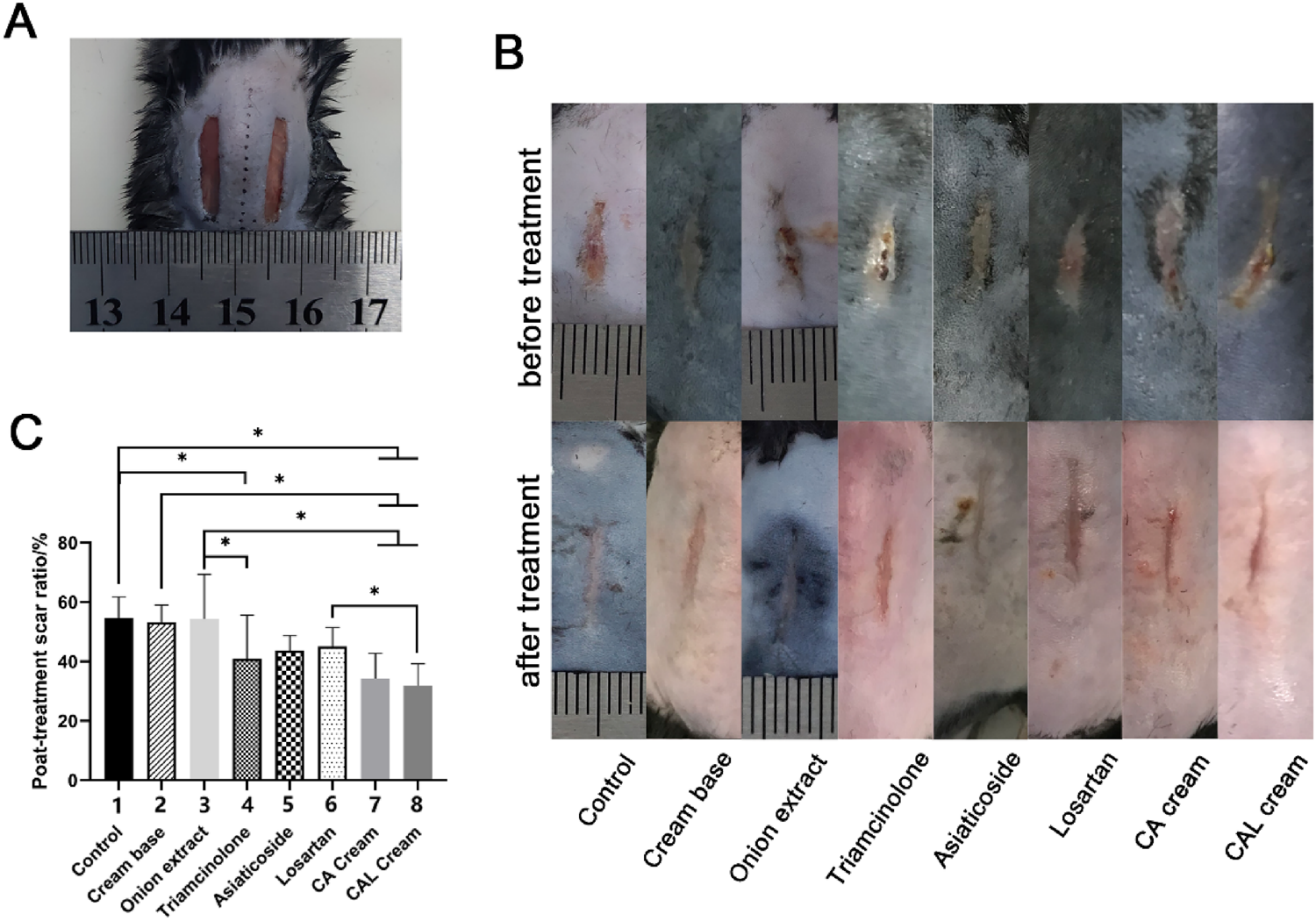
Scar observation in a murine scar model. (**A**) Establishment of mouse scar model. (**B**) Scars of the representative mice in each group on the 14th day (top) and the 28th day after surgery (bottom). (**C**) Statistical analysis of the shrinkage of the scar width. *P < 0.05 when group 1 versus 4, 7, and 8; group 2 versus 7 and 8; group 3 versus 4, 7, and 8; group 6 versus 8.

As shown in Fig. 2, the scar widths of the CAL cream and CA cream groups decreased significantly more than the control and cream base groups. The triamcinolone ointment had significantly more inhibition on scar formation compared to the control group; however, none of the test creams was better than the triamcinolone (*P* > 0.05). One of our aims is to develop a more efficient compound cream with better inhibitory effect on scarring than the losartan cream, and the CAL cream showed significant inhibition than the losartan cream on the macro level. But there was no significant difference between the onion extract gel group and the control group.

**Figure 3:**
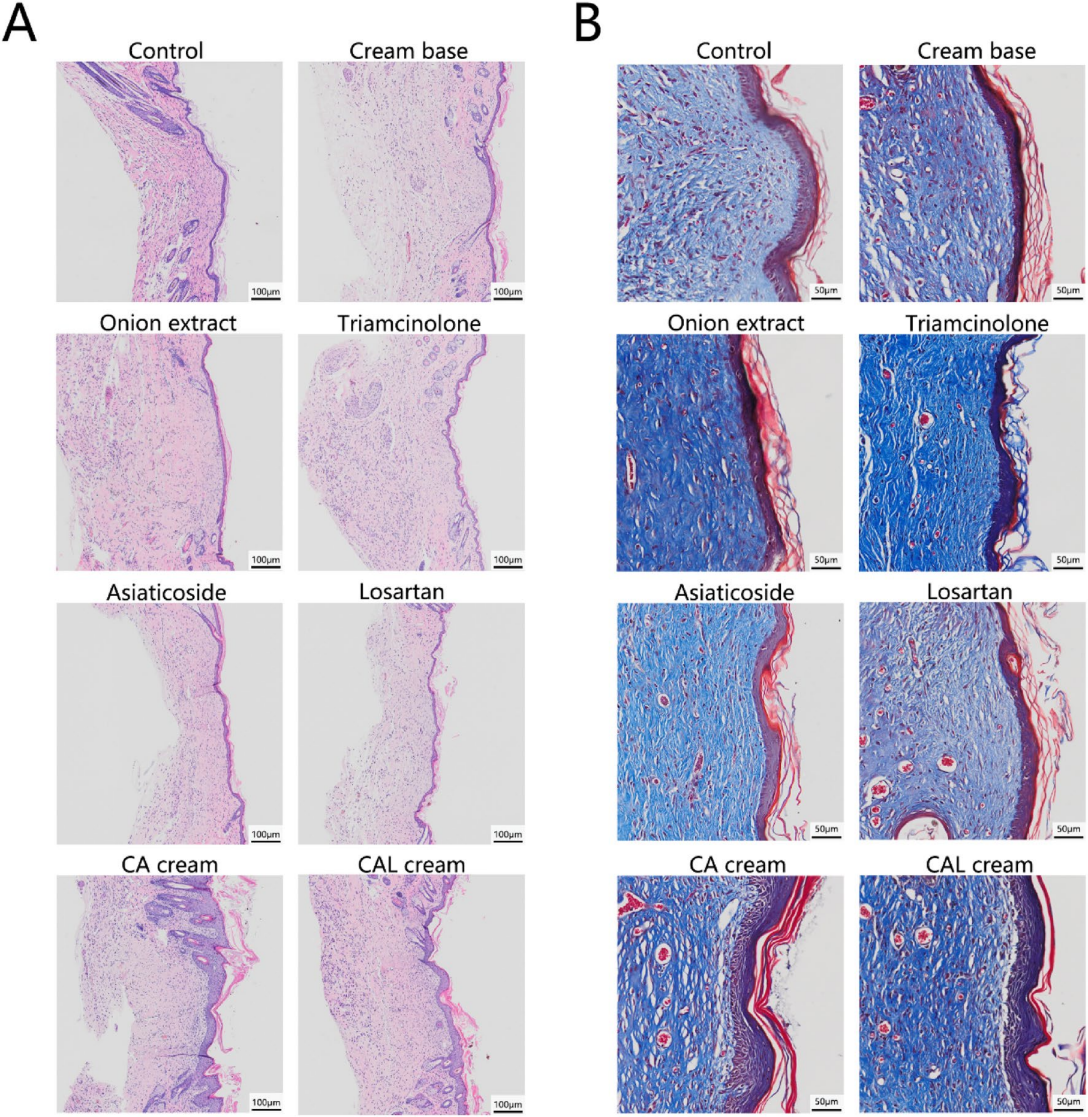
Typical histological sections of mouse scar tissue obtained on the 28th postoperative day. (**A**) HE staining. The scale bars = 100 μm. (**B**) Masson-trichrome staining. The scale bars = 50 μm.

**Figure 4:**
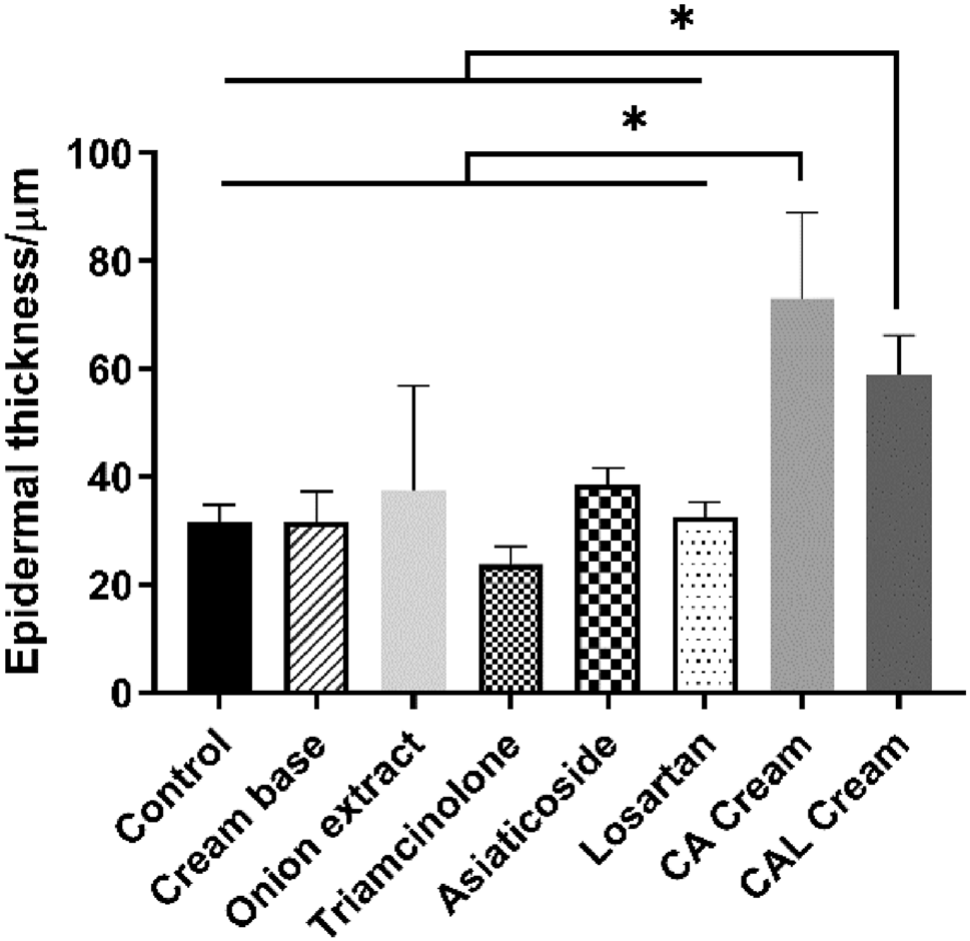
Epidermal thickness of groups. The epidermis of the CA cream group and the CAL cream group were significantly thicker than that of other groups.

The representative histological changes are shown in Fig. 3. The scars in the control and cream base groups were hyperplastic and elevated above the skin surface, while all scars treated with medicated creams were flatter. The epidermis of the CA cream group and the CAL cream group were significantly thicker than that of other groups (Fig. 4). The collagen fibers in the CA cream CAL cream group appeared to be distributed sparsely and arranged as a net-like shape. The arrangement of collagen fibers in the onion extract, triamcinolone, and asiaticoside groups also appeared to be more regular than those in the control and cream base groups, but the thickness of collagen fibers in the onion extract group did not appear to be as thin as those in other medical treated groups.

### Medicated creams inhibit TGF-β1 and collagen expression in scar

**Figure 5:**
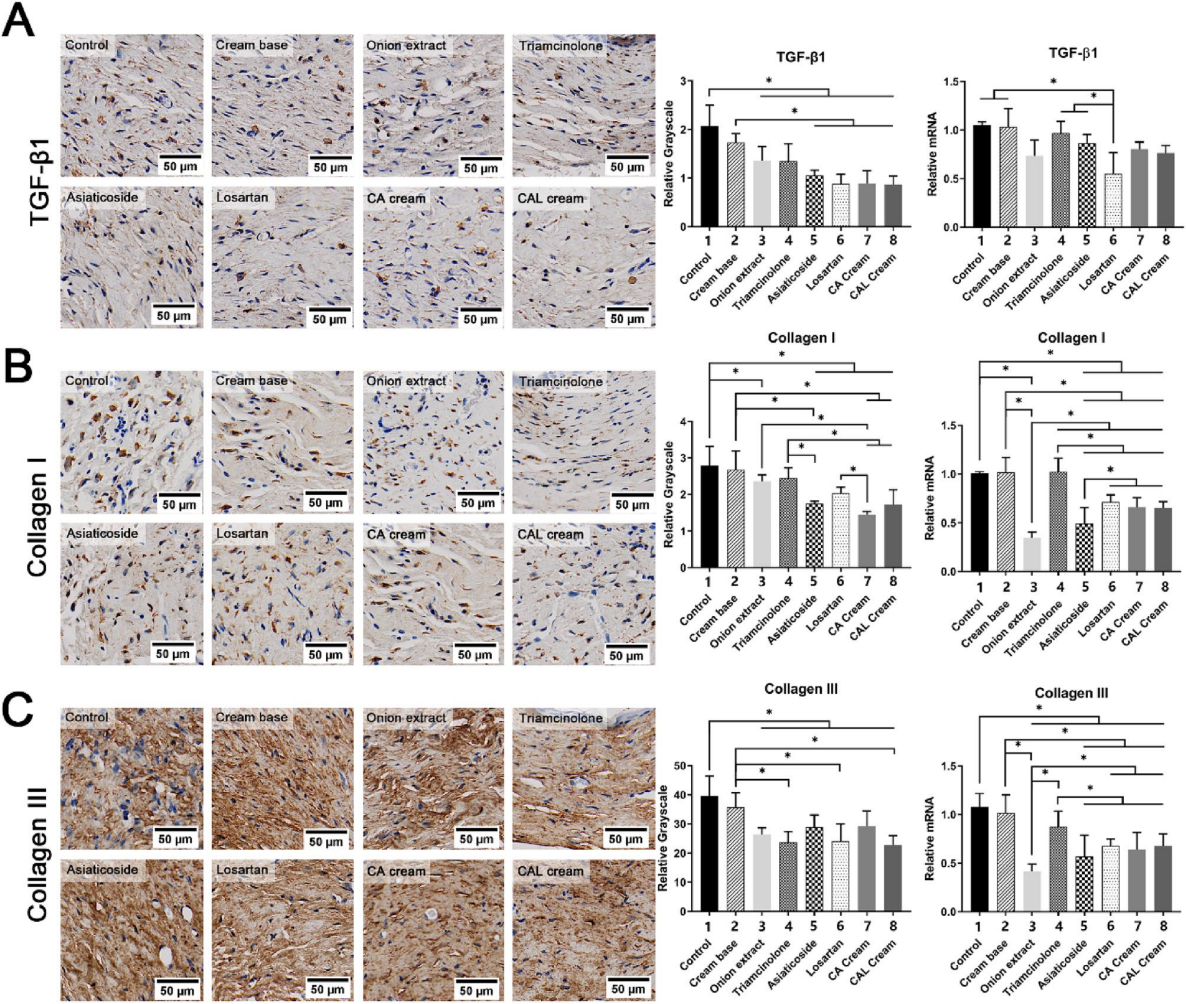
Medicated creams inhibit the expression of TGF-β1, collagen I, and collagen III in scar. (**A**) Immunohistochemical staining and RT-qPCR results of TGF-β1. IHC: *P < 0.05 when group 1 versus 3, 4, 5, 6, 7, and 8; group 2 versus 5, 6, 7, and 8. RT-qPCR: *P < 0.05 when group 6 versus 1, 2, 4, and 5. (**B**) Immunohistochemical staining and RT-qPCR results of collagen I. IHC: *P < 0.05 when group 1 versus 3, 5, 6, 7, and 8; group 2 versus 5, 7, and 8; group 3 versus 7; 4 versus 5, 7, and 8; group 6 versus 7. RT-qPCR: *P < 0.05 when group 1 versus 3, 5, 6, 7, and 8; group 2 versus 3, 5, 6, 7, and 8; group 3 versus 4, 5, 6, 7, and 8; group 4 versus 5, 6, 7, and 8; group 5 versus 6, 7, and 8. (**C**) Immunohistochemical staining and RT-qPCR results of collagen III. IHC: *P < 0.05 when group 1 versus 3, 4, 5, 6, 7, and 8; group 2 versus 4, 6, and 8. RT-qPCR: *P < 0.05 when group 1 versus 3, 4, 5, 6, 7, and 8; group 2 versus 3, 5, 6, 7, and 8; group 3 versus 4, 6, 7, and 8; group 4 versus 5, 6, 7, and 8. The scale bars = 50 μm. RT-qPCR results were normalized to GAPDH.

TGF-β1 is the core factor of scar formation, and its up-regulation leads to increased collagen expression. We compared TGF-β1 and collagen expression in scar tissue of each group at protein and mRNA levels by IHC and RT-qPCR, respectively. The statistical results are shown in Fig. 5.

The protein expression of TGF-β1 in all groups treated with medicated creams were significantly depressed compared to the control group. The three test creams we made showed more significant inhibition than the negative control (the cream base). However, only the losartan cream had inhibition on mRNA level compared to the control, the cream base, the triamcinolone, and the commercial asiaticoside cream. All medicated creams decreased the protein expression of collagen I compared with the control group except triamcinolone ointment, as well as the mRNA expression. The CA cream and the CAL cream showed more significant inhibition than the cream base and triamcinolone ointment. As for collagen III, all the groups treated with drugs had reduced expression of protein and mRNA, and the CAL cream group expressed significantly less than the cream base group.

### Medicated creams decrease the expression and phosphorylation of Smads in scar

To explore the further mechanism of the creams inhibiting the expression of TGF-β1 and collagen, we investigated the changes of TGF-β/Smad pathway.

**Figure 6:**
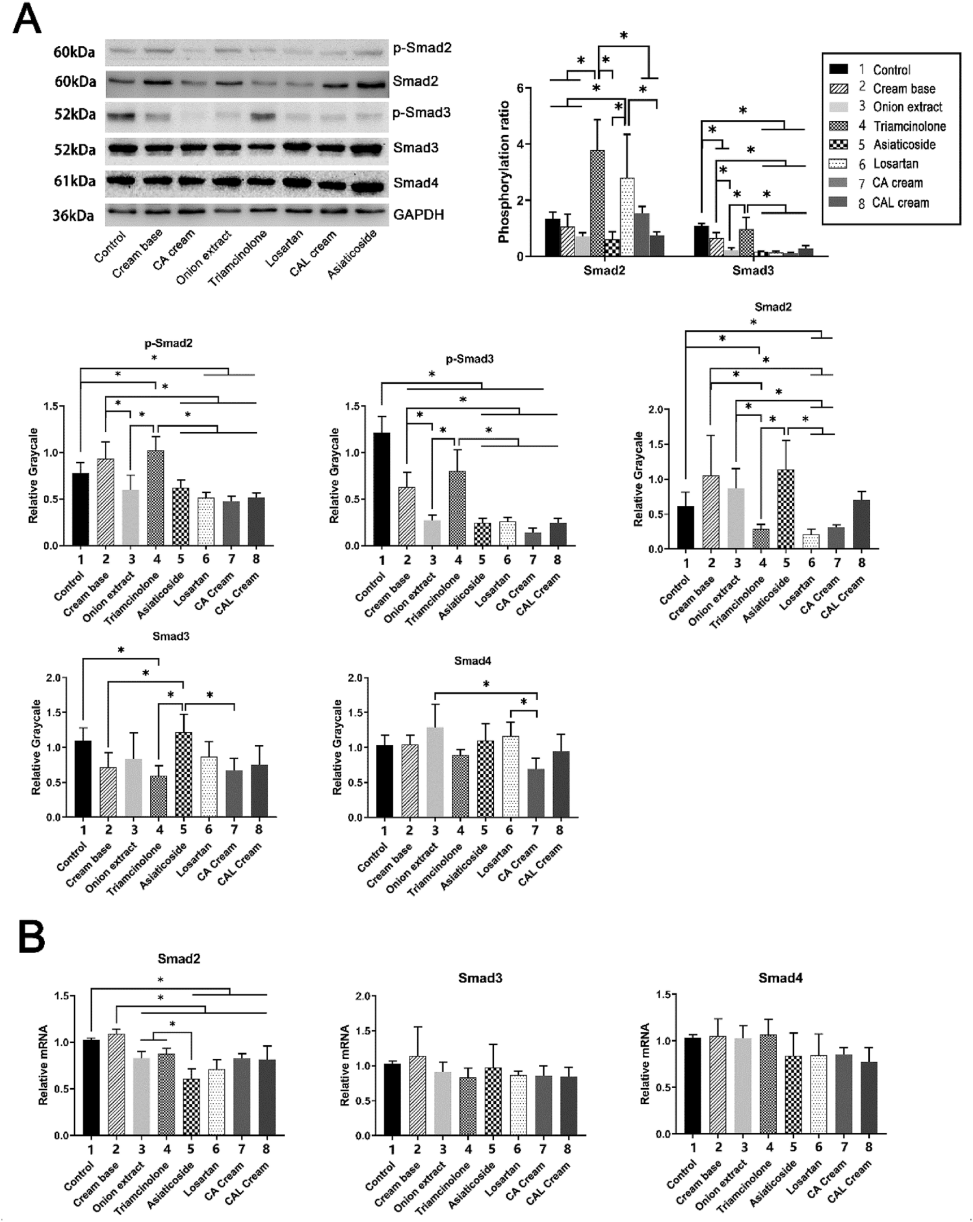
Medicated creams inhibit the expression and phosphorylation of Smads in scar. (**A**) Western blot results of p-Smad2, p-Smad3, Smad2, Smad3, and Smad4 in scar tissue, normalized to GAPDH. The western blot images were from different regions of different gels due to the similar molecular weight of the detected proteins, and all the western blot results were obtained under the same experimental conditions. P-Smad2 phosphorylation ratio: *P < 0.05 when group 4 versus 1, 2, 3, 5, 7, and 8; group 6 versus 1, 2, 3, 5, and 8. P-Smad3 phosphorylation ratio: *P < 0.05 when group 1 versus 2, 3, 5, 6, 7, and 8; group 2 versus 3, 5, 6, 7, and 8; group 4 versus 3, 5, 6, 7, and 8. P-Smad2: *P < 0.05 when group 1 versus 4, 6, 7, and 8; group 2 versus 3, 5, 6, 7, and 8; group 4 versus 3, 5, 6, 7, and 8. P-Smad3: *P < 0.05 when group 1 versus 2, 3, 4, 5, 6, 7, and 8; group 2 versus 3, 5, 6, 7, and 8; group 4 versus 3, 5, 6, 7, and 8. Smad2: *P < 0.05 when group 1 versus 4, 7, and 8; group 2 versus 4, 7, and 8; group 3 versus 4, 7, and 8; group 5 versus 4, 7, and 8. Smad3: *P < 0.05 when group 1 versus 4; group 2 versus 5; group 5 versus 4 and 7. Smad4: *P < 0.05 when group 3 versus 7; group 6 versus 7. (**B**) RT-qPCR results of Smad2, Smad3, and Smad4 in scar tissue, normalized to GAPDH. Smad2: *P < 0.05 when group 1 versus 5, 6, 7, and 8; group 2 versus 3 4, 5, 6, 7, and 8; group 5 versus 3 and 4. Smad3 and Smad4: no significant difference among groups. Unprocessed images of Western blots are presented in Supplementary Figures SF1.

According to Fig. 6, the groups treated with three test creams we made (the losartan cream, the CA cream, and the CAL cream) had significantly lower level of p-Smad2 and p-Smad3 than the control, cream base, and triamcinolone groups. These three test creams also showed inhibition of phosphorylation of Smad3. The phosphorylation of Smad2 in the CAL cream group was also diminished significantly compared to the triamcinolone and losartan cream groups.

As for the expression of the total Smad proteins (Smad2, Smad3, and Smad4), the losartan cream and the CA cream had significant inhibition on the protein and mRNA levels of Smad2. The CAL cream group also had decreased expression of Smad2 mRNA compared to the control and cream base groups, but there was no significant difference in Smad2 protein expression.

### TGF-β1 and collagen expression is attenuated by drugs in vitro

**Figure 7:**
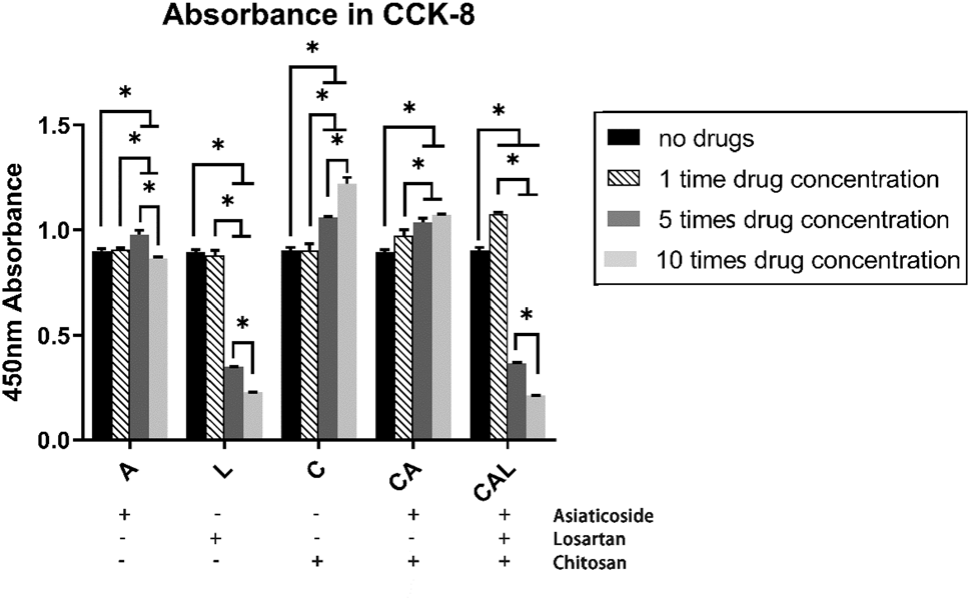
Cytotoxicity assay of drugs. A: group stimulated with *Centella asiatica* extract; L: group stimulated with losartan; C: group stimulated with mixture of chitosan; CA: group stimulated with mixture of chitosan and *Centella asiatica* extract; CAL: group stimulated with mixture of chitosan, *Centella asiatica* extract, and losartan. Drug concentrations were represented with multiples, single time drug concentration: 40 ng ml^−1^ mixture of chitosan, 50 ng ml^−1^ losartan, and 25 ng ml^−1^ *Centella asiatica* extract.

In the above experiments, we explored the effect of cream on the morphology of scar tissue and the expression of TGF-β/Smad pathway in the tissue. Scar formation has been proven to be closely related to fibroblast activity, so we next explored the effects of drugs in vitro with NIH3T3 cells. The concentrations of drugs we applied in the experiments in vitro were decided according to the concentrations contained in creams and the CCK-8 results (Fig. 7). Because losartan had significant inhibition on proliferation with concentration of 250 ng ml^−1^ and 500 ng ml^−1^, while 40 ng ml^−1^ mixture of chitosan, 50 ng ml^−1^ losartan, and 25 ng ml^−1^ *Centella asiatica* extract, whose main ingredient was asiaticoside, had no cytotoxicity to NIH3T3 cells, we chose these concentrations as the experimental ones.

**Figure 8:**
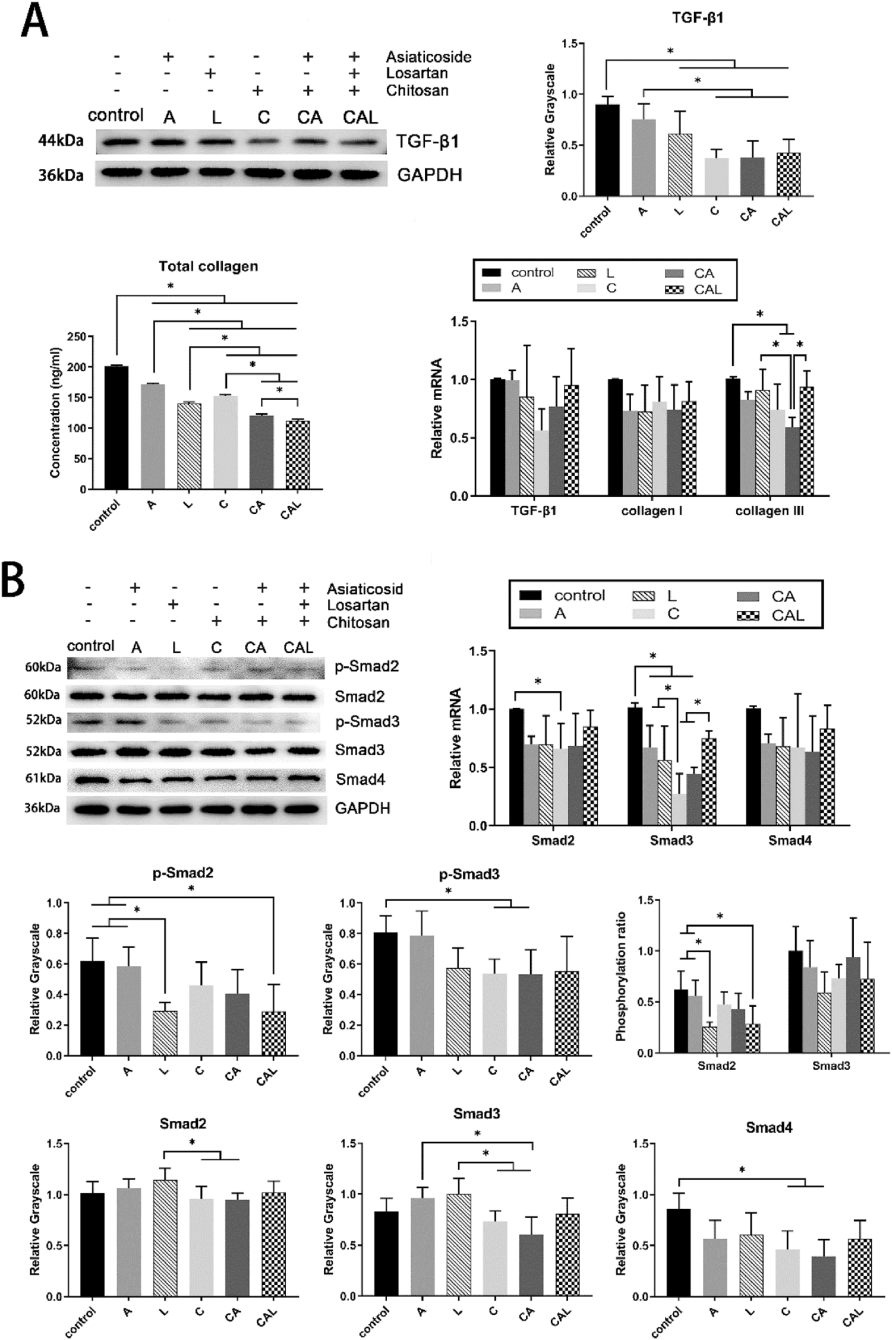
Drugs reduce the expression of TGF-β1 and collagen, inhibit TGF-β/Smad pathway in vitro. (**A**) Western blot result of TGF-β1 in NIH3T3 cells after drug stimulation, normalized to GAPDH; *P < 0.05 when control versus L, C, CA, and CAL; A versus C, CA, and CAL. The result of total collagen assay of cell culture medium; *P < 0.05 when all groups compared with each other. RT-qPCR results of TGF-β1, collagen I, and collagen III in NIH3T3 cells, normalized to GAPDH. TGF-β1 and collagen I: no significant difference among groups. Collagen III: *P < 0.05 when control versus C and CA; CA versus L and CAL. (**B**) Western blot results of p-Smad2, p-Smad3, Smad2, Smad3, and Smad4 in NIH3T3 cells, normalized to GAPDH. P-Smad2 phosphorylation ratio: *P < 0.05 when CAL versus control and A; L versus control and A. P-Smad3 phosphorylation ratio: no significant difference among groups. P-Smad2: *P < 0.05 when CAL versus control and A; L versus control and A. P-Smad3: *P < 0.05 when control versus C and CA. Smad2: *P < 0.05 when L versus C and CA. Smad3: *P < 0.05 when A versus CA; L versus C and CA. Smad4: *P < 0.05 when control versus C and CA. RT-qPCR results of Smad2, Smad3 in NIH3T3 cells, normalized to GAPDH. Smad2: *P < 0.05 when control versus C. Smad3: *P < 0.05 when control versus A, L, C, and CA; C versus A, and L; CAL versus C and CA. The western blot images were from different regions of different gels due to the similar molecular weight of the detected proteins, and all the western blot results were obtained under the same experimental conditions. Control: no drug stimulation; A: 25 ng ml^−1^ *Centella asiatica* extract; L: 50 ng ml^−1^ losartan; C: 40 ng ml^−1^ mixture of chitosan; CA: 40 ng ml^−1^ mixture of chitosan and 25 ng ml^−1^ *Centella asiatica* extract; CAL: 40 ng ml^−1^ mixture of chitosan, 25 ng ml^−1^ *Centella asiatica* extract, and 50 ng ml^−1^ losartan. TGF-β1 with 1 ng ml^−1^ concentration was added into the culture medium of all groups for injury simulation. Unprocessed images of Western blots are presented in Supplementary Figures SF2.

As shown in Fig. 8A, losartan and chitosan could reduce the expression of TGF-β1 protein. The three kinds of drugs we used (asiaticoside, losartan, and chitosan) had inhibition on the protein expression of collagen, and the inhibition was more obvious with combined use. As for the results of RT-qPCR, when chitosan was applied alone or combined with asiaticoside, the mRNA level of collagen III was significantly reduced.

In summary, losartan and chitosan could inhibit the expression of TGF-β1 and collagen protein, and the combined use had the same or even more significant effect, which was consistent with the IHC results of scar tissue.

### The drug combination inhibits the expression and phosphorylation of Smads of fibroblast in vitro

According to the results in vivo, creams showed inhibition on TGF-β/Smad signaling pathway. To verify the results in vivo, we analyzed the same pathway in the drug stimulation experiment with murine NIH3T3 fibroblasts.

As the results shown in Fig. 8B, the L group and CAL group had significantly decreased p-Smad2 expression and a lower phosphorylation ratio of Smad2. The C group and CA group had significantly decreased the p-Smad3 protein level; however, the addition of losartan did not have any positive effect. As for total Smad2 and Smad3, the inhibitions of the C and CA groups were more obvious compared with the L group, and the CA group had less Smad3 protein than the A group. Additionally, the Smad4 protein levels of the C group and CA group were lower than the control group. Compared with the control group, the C group had decreased level of Smad2 mRNA. The A, L, C, and CA groups also had lower levels of Smad3 mRNA than the control group. There was no significant difference in Smad4 mRNA level between groups.

These data indicated that losartan could inhibit the phosphorylation of Smad2. Chitosan and asiaticoside also had inhibition on Smad4, the co-Smad.

## Discussion

Intervention in the early stage is particularly important to prevent scar formation. The application of effective topical treatment is an alternative convenient anti-scar choice that can avoid the side effects of systemic medication. Therefore, the ultimate purpose of this study is to develop a cream with an effective anti-scarring effect.

In our previous study, we preliminarily developed losartan cream with an anti-scarring effect, but the inhibitory effect of it was not better than the triamcinolone ointment, the positive control^16^. Considering the anti-scarring effects of chitosan and asiaticoside involving TGF-β, we added these two kinds of drugs into losartan cream and improved the formula, to reach a better anti-scarring ability. As the results in vivo showed, the compound CAL cream decreased the scar width significantly more than the control, the cream base (the negative control), and the onion extract (one of the positive controls). Even though the inhibitory effect of the CAL cream was not significantly better than the triamcinolone ointment (the positive control in our previous study), importantly, the CAL cream decreased scar width more than the losartan cream, which was what we expected. In addition, the CA cream, which contained chitosan and *Centella asiatica* extract, also had remarkable anti-scarring ability. But the effect of the commercial onion extract gel, as one of the positive controls, was not as obvious as we expected (Fig. 2). It could only smooth the scar, but did not reduce the scar width. According to the histological results, scars treated with the CAL cream and the CA cream had sparsely distributed collagen fibers, and the arrangement of these fibers was net-like, which resembled normal skin (Fig. 3).

TGF-β1 overexpression and collagen deposition are prominent features of wound healing and scarring. Chitosan, asiaticoside, and losartan were all proven to have regulating effects on the expression of TGF-β1 and collagen^28,33,35,36^, and these inhibitory effects were verified in our study as well (Figs. 5, 8A).

It is worth mentioning that Baxter et al. reported different effects of chitosan on different periods of wound healing^36^. In the early post-injury period, chitosan could increase the expression of TGF-β1 and collagen III; During the later restoration phase, chitosan showed an inhibitory effect. In the drug stimulation experiment of our study, with 1 ng ml^−1^ TGF-β1 added into the medium for injury simulation, chitosan showed significant inhibition on the expression of TGF-β1 and collagen of NIH3T3 cells. We speculated that the concentration of TGF-β1 may affect the two-sided effect of chitosan.

Another interesting thing we found about chitosan in our experiments was that in the CCK-8 assay, chitosan showed a dose-dependent effect of promoting proliferation of NIH3T3 (Fig. 7). Patrulea et al. also proved that chitosan could promote the proliferation of HaCaT cells within 1 mg ml^−1^^23^. This similar phenomenon was also observed in our study. While both in vivo and in vitro experiments indicated that the use of chitosan could attenuate the expression of TGF-β1 and collagen (Figs. 5, 8A). Combined with the results of microscopic observation of scar tissue section, the epidermis of the CA cream group and the CAL cream group were significantly thicker (Fig. 3), and the morphological characteristics and distribution of collagen fibers were more similar to that of normal skin (Fig. 2). We could draw a conclusion that chitosan could accelerate the metabolism of the scar and the nearby tissues, and it could also inhibit collagen production but promote regular arrangement of collagen fibers. These properties of chitosan also contributed to the best inhibitory effect of the CAL cream on scar formation.

The reduction of TGF-β1 expression caused by these drugs decreased the expression of collagen, resulting inhibitory effect on scar formation. TGF-β/Smad is a classical pathway downstream of TGF-β1 to regulate fibrosis^37^. Therefore, we next set our sights on this signaling pathway to further explore the mechanism of the anti-scarring effect of creams. We found that the CAL cream had significant inhibition on TGF-β/Smad pathway, especially on the phosphorylation of Smad2 and Smad3 (Fig. 6). These inhibitory effects on the scar tissue were the combined effects of losartan, chitosan, and asiaticoside.

In order to explore the specific effects of each drug on TGF-β/Smad signaling pathway in fibroblast, we next performed further investigation with NIH3T3 fibroblasts in vitro.

Angiotensin II can increase the expression of TGF-β1 and Smad^38,39^, it could also induce the phosphorylation of Smad2 and the activation of TGF-β1^39^, and the regulation is mediated by the AT1 receptor^40^. Therefore, the inhibitory effect of RAS inhibitor on TGF-β/Smad pathway has been widely recognized. In our study, the inhibitory effect of losartan on TGF-β/Smad pathway was also remarkable (Fig. 8). Lim et al. reported the inhibitory effects of chitosan on the expression of TGF-β1, Smad2, and Smad4, which was useful in keloid intervention^41^. As shown in Fig. 8, chitosan showed distinctive inhibition on the protein expression of p-Smad3 as well as Smad4, and it had negative effects on the mRNA expression of Smad2 and Smad3. As for asiaticoside, Tang et al. had also found that asiaticoside had negative regulation on the expression of TGF-βRI and TGF-βRII, and it could enhance the expression of Smad7. However, p-Smad2, p-Smad3, Smad2, Smad3, and Smad4 were not significantly inhibited in their study as well^28^. In our study, asiaticoside showed inhibition on the levels of collagen and the Smad3 mRNA, but there was no other significant inhibition of Smads protein or mRNA.

**Figure 9:**
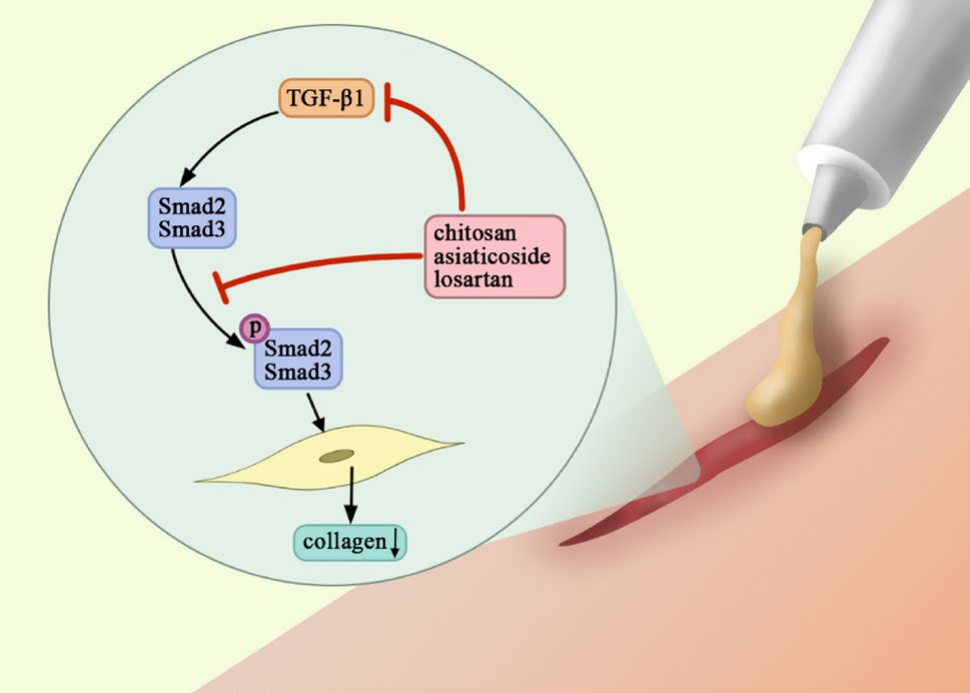
The compound losartan cream could inhibit scar formation via TGF-β/Smad pathway.

Combining the results of the cream experiment in vivo and the drug stimulation in vitro, we could draw the conclusion that the best inhibitory effect on scar formation of the CAL cream at least partly results from the inhibition on TGF-β/Smad signaling pathway (Fig. 9).

As a convenient scar treatment method with great medication compliance, topical ointments have great potential for development. The compound losartan cream (the CAL cream) we made was suggested to be an option worth considering because of the excellent anti-scarring effect, and we elucidated that the inhibition on TGF-β/Smad pathway contributed to the anti-scarring effect of the CAL cream. There have been a lot of potential scar therapies targeting TGF-β/Smad pathway^42^, however, these therapies are still in the stage of preclinical research. Considering the practical application of the topical scar treatment ointments, clinical trials are essential, and this will be the direction of our next efforts.

## Conclusions

In summary, we developed a compound losartan cream (the CAL cream) with a significant inhibitory effect on scarring. An exploration of the mechanism showed that the inhibition on TGF-β/Smad signaling pathway contributed to the effect of the CAL cream on scar inhibition.

## Supplementary Information

Supplementary Information.

## Data Availability

The datasets used and/or analyzed during the current study available from the corresponding author on reasonable request.

## Abbreviations

ARB: Angiotensin receptor blocker
ACEI: Angiotensin converting enzyme inhibitor
CA cream: Chitosan asiaticoside cream
CAL cream: Compound losartan cream
DMEM: Dulbecco’s Modified Eagle Medium
FITC: Fluorescein isothiocyanate
HE: Hematoxylin-eosin
HPLC: High performance liquid chromatography
IHC: Immunohistochemistry
RT-qPCR: Reverse transcription-quantitative polymerase chain reaction
PVDF: Polyvinylidene fluoride
SDS-PAGE: Sodium dodecyl sulphate–polyacrylamide gel electrophoresis

## References

[1] Brown B, McKenna S, Siddhi K, McGrouther D, Bayat A (2008). The hidden cost of skin scars: Quality of life after skin scarring. J. Plast. Reconstr. Aesthet. Surg..

[2] Gauglitz GG, Korting HC, Pavicic T, Ruzicka T, Jeschke MG (2011). Hypertrophic scarring and keloids: Pathomechanisms and current and emerging treatment strategies. Mol. Med..

[3] Fang QQ (2017). The effectiveness of topical anti-scarring agents and a novel combined process on cutaneous scar management. Curr. Pharm. Des..

[4] Thomas CR (2012). Personality disorders in young adult survivors of pediatric burn injury. J. Pers. Disord..

[5] Tullington JE, Gemma R (2021). 2021 StatPearls.

[6] Thomas JR, Somenek M (2012). Scar revision review. Arch. Facial Plast. Surg..

[7] Marshall CD (2018). Cutaneous scarring: Basic science, current treatments, and future directions. Adv. Wound Care.

[8] González N, Goldberg DJ (2019). Update on the treatment of scars. J. Drugs Dermat..

[9] Wu LL (1997). Transforming growth factor β1 and renal injury following subtotal nephrectomy in the rat: Role of the renin-angiotensin system. Kidney Int..

[10] Unger T (2002). The role of the renin–angiotensin system in the development of cardiovascular disease. Am. J. Cardiol..

[11] Bataller R, Sancho-Bru P, Ginês P, Brenner DA (2005). Liver fibrogenesis: A new role for the renin–angiotensin system. Antioxid. Redox Signal..

[12] Steckelings UM (2004). Human skin: source of and target organ for angiotensin II. Exp. Dermatol..

[13] Morihara K (2006). Cutaneous tissue angiotensin-converting enzyme may participate in pathologic scar formation in human skin. J. Am. Acad. Dermatol..

[14] Rosenkranz S (2004). TGF-β1 and angiotensin networking in cardiac remodeling. Cardiovasc. Res..

[15] Tan WQ (2018). Angiotensin-converting enzyme inhibitor works as a scar formation inhibitor by down-regulating Smad and TGF-β-activated kinase 1 (TAK1) pathways in mice. Br. J. Pharmacol..

[16] Zheng B (2019). The effect of topical ramipril and losartan cream in inhibiting scar formation. Biomed. Pharmacother..

[17] Azad AK, Sermsintham N, Chandrkrachang S, Stevens WF (2004). Chitosan membrane as a wound-healing dressing: Characterization and clinical application. J. Biomed. Mater. Res. Part B Appl. Biomater..

[18] Okamoto Y (1993). Application of polymeric N-acetyl-D-glucosamine (chitin) to veterinary practice. J. Vet. Med. Sci..

[19] Dai T, Tanaka M, Huang Y-Y, Hamblin MR (2011). Chitosan preparations for wounds and burns: Antimicrobial and wound-healing effects. Expert Rev. Anti Infect. Ther..

[20] Shukla A (1999). In vitro and in vivo wound healing activity of asiaticoside isolated from *Centella asiatica*. J. Ethnopharmacol..

[21] Zhang CZ (2016). Porous microspheres as promising vehicles for the topical delivery of poorly soluble asiaticoside accelerate wound healing and inhibit scar formation in vitro & in vivo. Eur. J. Pharmaceut. Biopharmaceut..

[22] Senel S, McClure SJ (2004). Potential applications of chitosan in veterinary medicine. Adv. Drug Deliv. Rev..

[23] Patrulea V, Ostafe V, Borchard G, Jordan O (2015). Chitosan as a starting material for wound healing applications. Eur. J. Pharmaceut. Biopharmaceut..

[24] Ribeiro MP (2009). Development of a new chitosan hydrogel for wound dressing. Wound Repair Regen..

[25] Hu S (2018). Preparation of composite hydroxybutyl chitosan sponge and its role in promoting wound healing. Carbohydr. Polym..

[26] Yun K-J (2008). Inhibition of LPS-induced NO and PGE2 production by asiatic acid via NF-κB inactivation in RAW 2647 macrophages: Possible involvement of the IKK and MAPK pathways. Int. Immunopharmacol..

[27] Guo JS, Cheng CL, Koo MWL (2004). Inhibitory effects of Centella asiatica water extract and asiaticoside on inducible nitric oxide synthase during gastric ulcer healing in rats. Planta Med..

[28] Tang B (2011). Asiaticoside suppresses collagen expression and TGF-β/Smad signaling through inducing Smad7 and inhibiting TGF-βRI and TGF-βRII in keloid fibroblasts. Arch. Dermatol. Res..

[29] Tan, W. Q. & Yang, H. Chitosan skin care liquid and preparation method. *China Nation Patent*.

[30] Yang H, Zheng L, Huang X, Zhang M, Tan W (2011). Chitosan liquid improves wound healing in rats. Chin. J. Dermatol..

[31] Song M, Xin Z (2005). Assay and test for related substances of Losartan potassium by HPLC. West China J. Pharmaceut. Sci..

[32] Yang, M. *et al.* Determination of madecassoside and asiaticoside in Centella asiatica formula granules by HPLC method. *J. Pharmaceut. Pract.* 359–361 (2017).

[33] Fang QQ (2018). Angiotensin-converting enzyme inhibitor reduces scar formation by inhibiting both canonical and noncanonical TGF-beta1 pathways. Sci. Rep..

[34] Aranaz I (2018). Cosmetics and cosmeceutical applications of chitin, chitosan and their derivatives. Polymers.

[35] Murphy A (2019). Angiotensin II type I receptor blockade is associated with decreased cutaneous scar formation in a rat model. Plast. Reconstr. Surg..

[36] Baxter RM (2013). Chitosan dressing promotes healing in third degree burns in mice: Gene expression analysis shows biphasic effects for rapid tissue regeneration and decreased fibrotic signaling. J. Biomed. Mater. Res. Part A.

[37] Huang C, Akaishi S, Ogawa R (2012). Mechanosignaling pathways in cutaneous scarring. Arch. Dermatol. Res..

[38] Campbell SE, Katwa LC (1997). Angiotensin II stimulated expression of transforming growth factor-β1in cardiac fibroblasts and myofibroblasts. J. Mol. Cell. Cardiol..

[39] Hao J, Wang B, Jones SC, Jassal DS, Dixon IM (2000). Interaction between angiotensin II and Smad proteins in fibroblasts in failing heart and in vitro. Am. J. Physiol. Heart Circulat. Physiol..

[40] Bomb R (2016). Myofibroblast secretome and its auto-/paracrine signaling. Expert Rev. Cardiovasc. Ther..

[41] Lim CK, Halim AS, Yaacob NS, Zainol I, Noorsal K (2013). Keloid pathogenesis via Drosophila similar to mothers against decapentaplegic (SMAD) signaling in a primary epithelial-mesenchymal in vitro model treated with biomedical-grade chitosan porous skin regenerating template. J. Biosci. Bioeng..

[42] Zhang T (2020). Current potential therapeutic strategies targeting the TGF-β/Smad signaling pathway to attenuate keloid and hypertrophic scar formation. Biomed. Pharmacother..

