# Supplementary material for: The compound losartan cream inhibits scar formation via TGF-β/Smad pathway
Source: Sci Rep. 2022 Aug 22;12:14327. doi: 10.1038/s41598-022-17686-y (PMC9395380; doi:10.1038/s41598-022-17686-y)

Title page

Title: The compound losartan cream inhibits scar formation via TGF-β/Smad pathway

Author List: Wan-Yi Zhao, M.D[1, *]; Li-Yun Zhang, M.D[1, *]; Zheng-Cai Wang, M.D[1]; Qing-Qing Fang, Ph. D[1]; Xiao-Feng Wang, Ph. D[1]; Yong-Zhong Du, Ph. D[3,1]; Bang-Hui Shi, M.M[4]; Dong Lou, Ph.D[1]; Gui-Da Xuan, M.M[2,#]; Wei-Qiang Tan, M.D[1,#]

1 Department of Plastic Surgery, Sir Run Run Shaw Hospital, Zhejiang University School of Medicine, Hangzhou, Zhejiang Province, P.R. China.

2 School of Medicine, Zhejiang University City College, Hangzhou, Zhejiang Province, P.R. China.

3 Institute of Pharmaceutics, College of Pharmaceutical Sciences, Zhejiang University, Hangzhou, Zhejiang Province, P.R. China.

4 Department of Plastic Surgery, The Fourth Affiliated Hospital, Zhejiang University School of Medicine, Yiwu, Zhejiang Province, P.R. China.

* These two authors contributed equally to this work.

# These two corresponding authors contributed equally to this work.

Supplementary Materials

Supplementary Figure 1. Creams inhibit the expression and phosphorylation of Smads in vivo.

Unprocessed Western blot of scar tissue from Figure 6A.

SF1

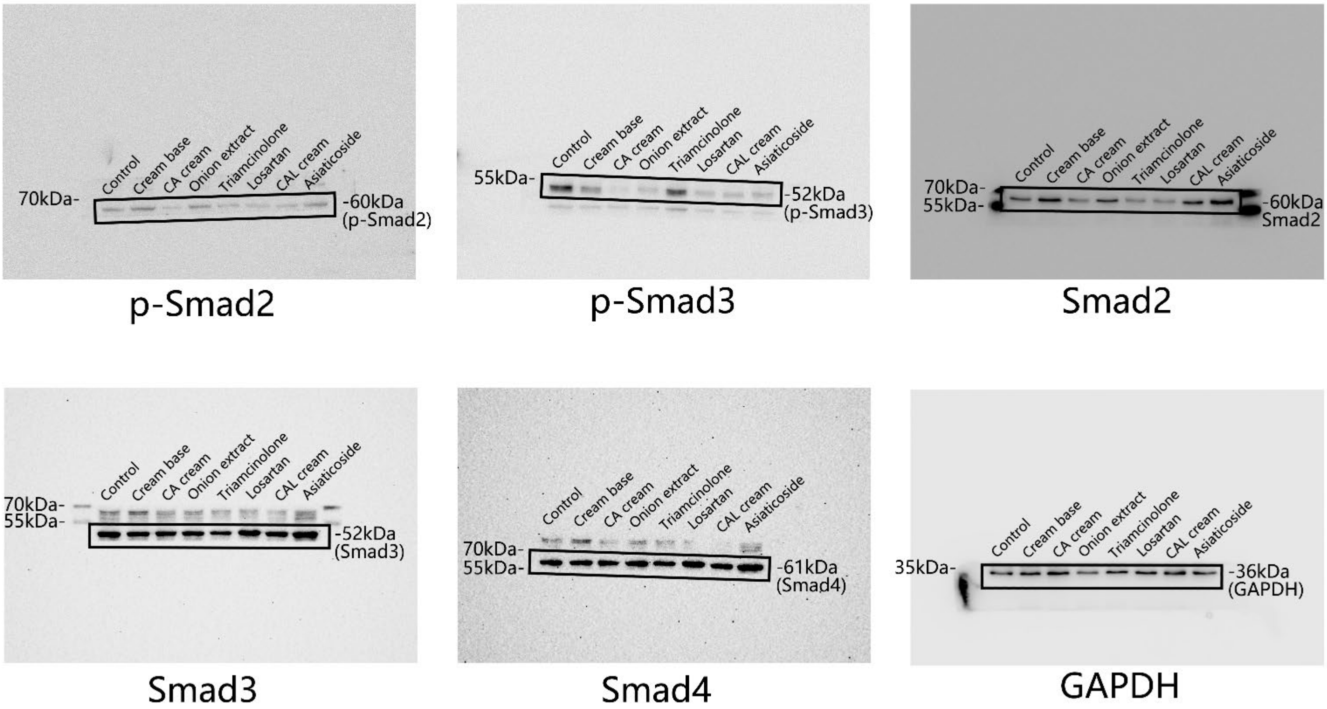

Supplementary Figure 2. Drugs reduce the expression of TGF-β1 and collagen, inhibit TGF-β/Smad pathway in vitro.

A. Unprocessed Western blot of scar tissue from Figure 8A.

B. Unprocessed Western blot of scar tissue from Figure 8B.

SF2

A

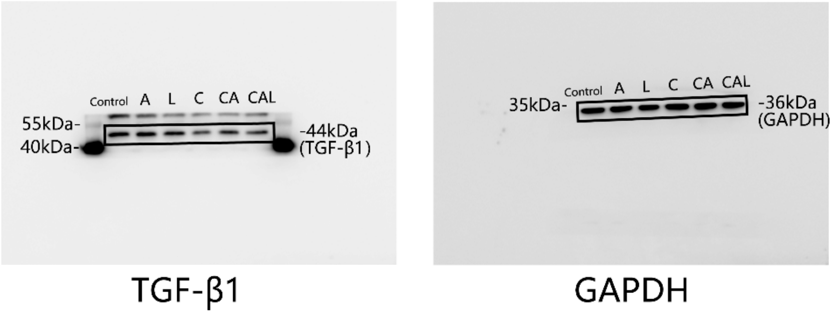

B

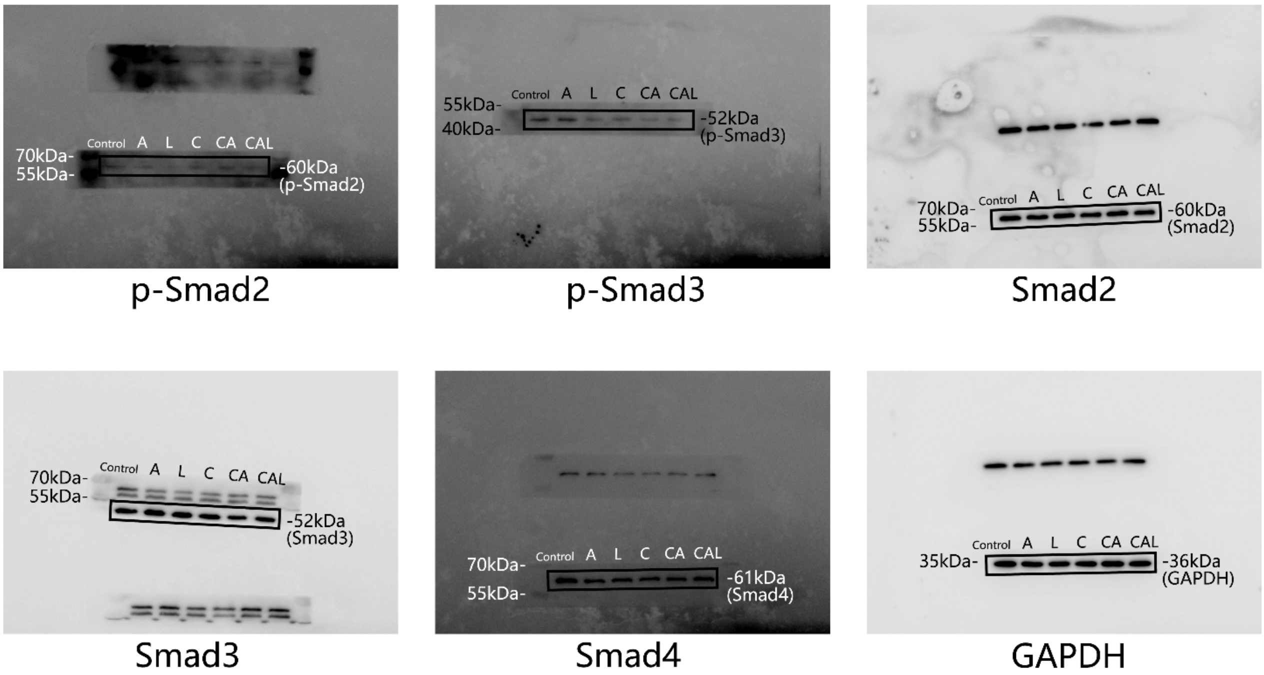

Supplement: Supplementary file 1 — Supplementary Information. [file 41598_2022_17686_MOESM1_ESM.pdf]
